# Case Report: Novel *ADA2* variants cause atypical adenosine deaminase 2 deficiency

**DOI:** 10.3389/fgene.2024.1478581

**Published:** 2025-01-15

**Authors:** Haishao Yu, Shuangzhu Lin, Lin Li, Jiayi Li, Qiandui Chen, Yuheng Wu, Yangfan Qi, Wanqi Wang, Xingzhi Chang, Jie Zhang

**Affiliations:** ^1^ Department of Pediatrics, Yantai Yuhuangding Hospital, Shandong, China; ^2^ Department of Pediatrics, Peking University First Hospital, Beijing, China; ^3^ Department of Pediatrics, First Affiliated Hospital to Changchun University of Chinese Medicine, Jilin, China; ^4^ Department of Pediatrics, The Third Affiliated Hospital of Zhengzhou University, Zhengzhou, China; ^5^ Department of Pediatrics, Changchun University of Chinese Medicine, Jilin, China; ^6^ Clinical Medical College, Norman Bethune Health Science Center of Jilin University, Changchun, China

**Keywords:** deficiency of adenosine deaminase 2, *ADA2* gene, *CECR1 gene*, children, hemiplegia

## Abstract

**Case presentation:**

A girl aged 2 years and 5 months presented to the hospital with chief complaints of intermittent fever and weakness of the left limb for more than 1 month. The child had transient urticaria appearing on her face for 5 days. The inflammatory biomarkers were significantly increased. Brain MRI showed multiple ischemic lesions in the brain’s small vessels. The patient exhibited significant systemic inflammation and multiple vasculitis. Whole-exome sequencing showed c.1358A>G p. (Tyr453Cys) and c.1082-7T>A compound heterozygous variants in the adenosine deaminase 2 (*ADA2*) gene, of which the c.1082-7T>A variant has not been reported yet in previous literature. Peripheral blood mRNA reverse transcription-Sanger sequencing confirmed that this variant affected mRNA splicing, resulting in a frameshift with premature stop codon c.1083_1103del p. (Leu362Glnfs*45). Peripheral blood test suggested a significant decrease in ADA2 activity. Eventually, the patient was diagnosed with deficiency of adenosine deaminase 2 (DADA2). Her condition improved after treatment with etanercept. She had no more fevers, and no hemiplegia attacks were observed during the 3 years of follow-up.

**Conclusion:**

Fever and hemiplegia were the main manifestations in this patient, without typical rashes. DADA2 was finally confirmed by enzymology and genetic testing, and we believe this is the first reported case of the c.1082-7T>A intronic variant in DADA2, and the RNA studies conducted in this case have been pivotal in assessing its pathogenicity.

## 1 Introduction

The *ADA2* gene encodes adenosine deaminase 2 (OMIM#607575) and is located on chromosome 22q11.1, with a total length of about 30 kb. It comprises nine exons and contains an open reading frame of 1,536 bp (Riazi et al., 2000). The most common genotype for affected individuals is characterized by compound heterozygosity for missense pathogenic variants. The more frequently observed variants are c.139G>A p. (Gly47Arg), c.140G>C p. (Gly47Ala), c.506G>A p. (Arg169Gln) and c.1358A>G p. (Tyr453Cys) (Zhou et al., 2014; Navon et al., 2014). Deficiency of adenosine deaminase 2 (DADA2, MIM#615688) is a rare autoinflammatory disease caused by variant of the *ADA2* gene (also known as *CECR1* gene) (Navon et al., 2014). DADA2 follows the autosomal recessive pattern of inheritance. Vasculopathy, immunodeficiency and bone marrow failure are its typical manifestations. It has complex and diverse manifestations and often involves multiple systemic manifestations. DADA2 can be easily misdiagnosed, and early diagnosis is crucial for good prognosis.

At present, there are more than 316 reported cases of DADA2 in literature, and most of them are pediatric patients. More than half the patients have early childhood onset, with >80% having disease onset before the age of 18 months (Pinto et al., 2021). Further, 90% cases reported skin involvement, and the typical skin vascular lesions are livedo reticularis and erythema nodules (Lee, 2018). Herein, we report a case of a 2.5-year-old girl affected by DADA2, whose presenting symptoms were fever and hemiplegia, without typical dermatological findings, bearing the c.1358A>G, p. (Tyr453Cys) and c.1082-7T>A variants in compound heterozygosity. The c.1082-7T>A variant has not yet been reported in the literature. We believe our report has enriched the genetic spectrum of DADA2.

## 2 Case description

A 2.5-year-old girl with the main complaints of intermittent fever and left-limb weakness for more than 1 month was admitted in Peking University First Hospital. She had fever without obvious symptoms of infection. At the beginning of the disease, the fever was low-to-moderate, but it subsequently became high (39°C–41°C), without any other infectious symptoms. Left-limb weakness appeared after 2 weeks of disease onset, which peaked in 3 days. The muscle strength of the left limb was grade 0, with left-sided central facial palsy. Various antibiotics and antiviral drugs such as ceftriaxone, cefoperazone-sulbactam, azithromycin, meropenem, vancomycin, fluconazole, and acyclovir, in addition to gamma globulin were confirmed to be ineffective. She had delayed intellectual and motor development. She raised her head at 4 months, sat without support at 9 months, and walked without support at the age of 2 years. In addition, she could only speak short sentences with a maximum of 3-4 words at a time. Mild anemia was found at the age of 1 year, and iron supplementation showed no improvement of symptoms. A transient left eyelid ptosis occurred at the age of 1 year and 5 months, but symptoms get better on their own after a week. The patient was conscious at first presentation. Her height and weight were 85 cm (P3-10) and 13.2 kg (P50-75), respectively, and her head circumference was 47.5 cm (P25-50). No rashes or superficial lymph nodes were observed. Cardiopulmonary and abdominal examination showed no abnormalities. Left-sided central facial palsy was observed, and muscle strength of the left limbs were grade III. Muscle tone was increased on the left limbs. Bilateral knee tendons showed hyperreflexia. The left ankle clonus was positive, and Babinski signs were negative involving both sides.

**FIGURE 1:**
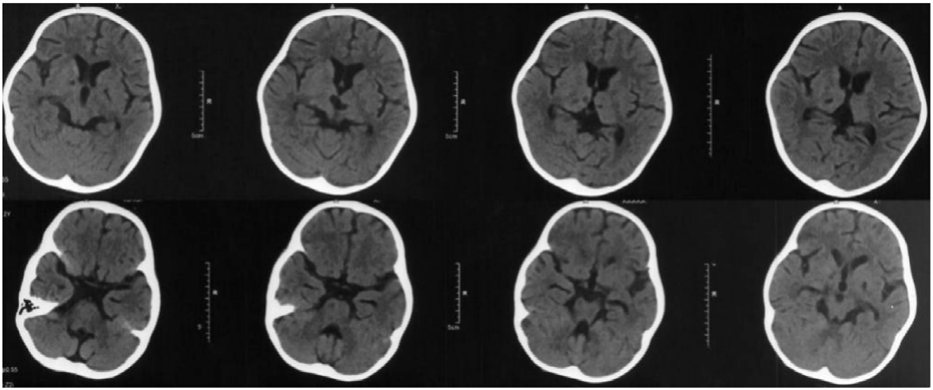
Brain CT taken at 2 years and 4 months old, and 2 weeks after disease onset: multiple spotty and patchy low-density lesions in the bilateral basal ganglia and thalamus.

The count of white blood cell and platelets were normal. Hemoglobin was 94 g/L. C-reactive protein (CRP) was 76 mg/L (normal range 0-8 mg/L) and erythrocyte sedimentation rate (ESR) was 47 mm/h (normal range 0-20 mm/h). IgA 0.19 g/L (0.31–0.67 g/L), IgM 0.61 g/L (0.98–1.789 g/L) and C3 1.71 g/L (0.6–1.5 g/L) were abnormal. IgG and C4 were normal. Serum autoimmune antibodies and demyelinating related antibodies (including Myelin Oligodendrocyte Glycoprotein-IgG and Aquaproin Protein-4-IgG) were negative. White blood cells, protein and glucose in cerebrospinal fluid were normal. Bone marrow morphologic examination was also normal. Brain computed tomography (CT) showed multiple spotty and patchy low-density lesions in bilateral basal ganglia and thalamus (Figure 1).

**FIGURE 2:**
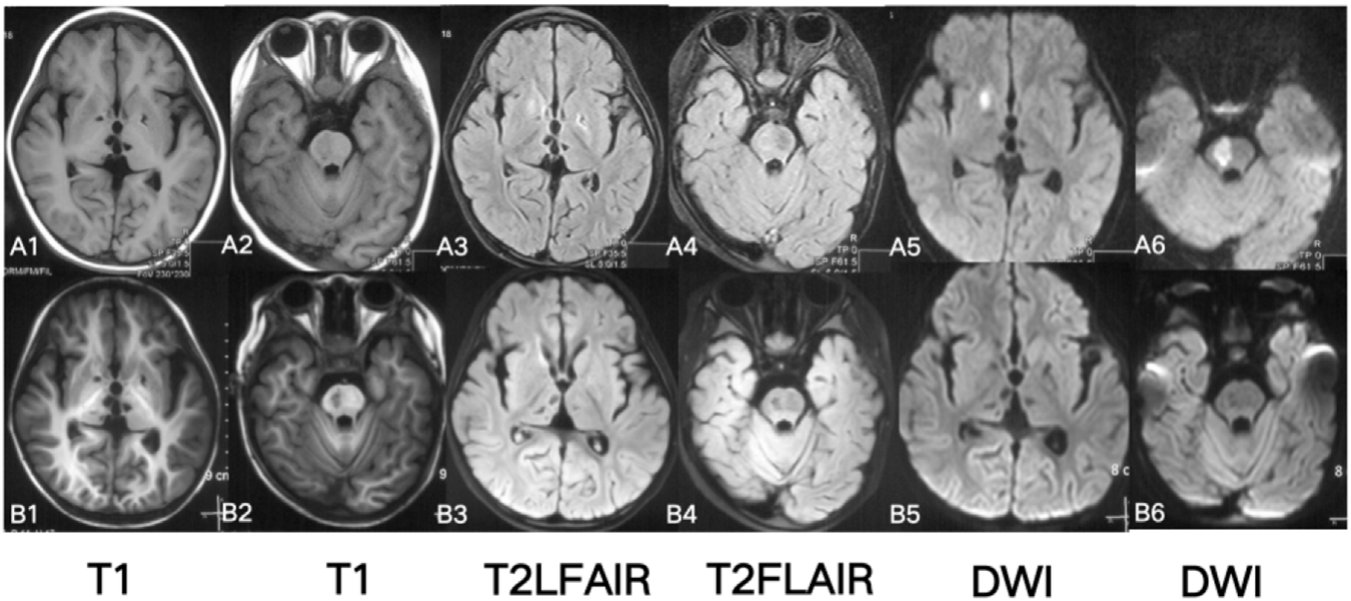
Brain MRI A1-6: at 2 years and 4 months of age (2 weeks of disease duration after disease onset): T2WI, T2-FLAIR, DWI high, and T1WI low signals in the right putamen and brainstem; T2WI, T2-FLAIR, and T1WI low signals in the bilateral thalamus and basal ganglia. B1-6: at 2 years and 5 months old (1.5 months after disease onset): T2, T2-FLAIR high signals in the right putamen and right brainstem disappeared.

Brain Magnetic Resonance Imaging (MRI) showed high signal in right putamen and right brainstem on T2-FLAIR (Fluid attenuated inversion recovery) and DWI (Diffusion-weighted imaging). The bilateral basal ganglia and thalamus were patchy with low signal on T1, low signal on T2-FLAIR, and DWI (Figure 2). No abnormality was found on head Magnetic Resonance Angiography (MRA) and spinal cord MRI.

**FIGURE 3:**
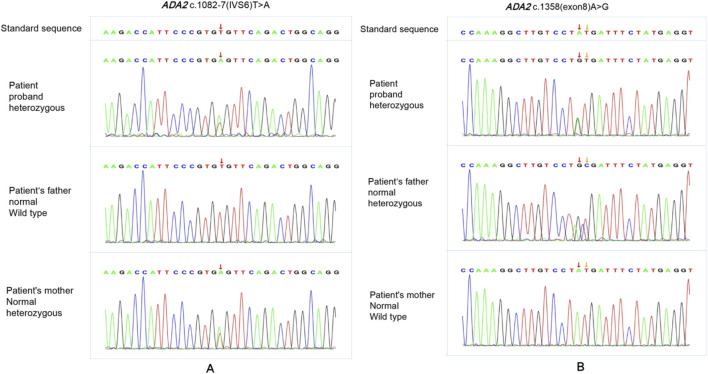
The Sanger sequencing of the patient’s family. The patint had compound heterozygous variants in *ADA2* gene, NM_017424 c.1358A>G p. (Tyr453Cys)and c.1082-7T>A. The variant NM_017424 c.1082-7T>A was found in both the patient and her mother **(A)**. The variant NM_017424 c.1358A>G p. (Tyr453Cys) was found in both the patient and her father **(B)**.

The patient showed persistent fever and hemiplegia with acute onset. The CRP and ESR levels were significantly increased, showing significant systemic inflammation. Brain MRI showed multiple ischemic lesions in the brain’s small vessels. Some were new lesions and some were pre-existing ones. We initially thought it was systemic vasculitis. However, the early onset of her condition prompted us to reassess the diagnosis, and we considered the possibility of hereditary vasculitis—a monogenic disorder characterized by skin and organ vasculitis as the primary clinical manifestation, which may be caused by gene variants. Next, the patient and her parents underwent trio Whole Exome Sequencing (WES; Beijing Zhiyin Oriental Translational Medicine Research Center) to confirm the diagnosis. Informed consent was obtained from her parents, and peripheral blood was collected for WES. The compound heterozygous variants in the *ADA2* gene were identified through the trio-WES and Sanger sequencing (Figure 3).

Variant 1 is c.1082-7T>A (Figure 3A): This intron variation had not been recorded in gnomAD (version 4.1.0), 1,000 genome Project, dbSNP or Clinvar Database (PM2_Supporting). MaxEntScan and spliced_bscSNV software predicted the variant to be deleterious (PP3_moderate). The variant was classificated as VUS according to the American College of Medical Genetics and Genomics (ACMG) guidelines (PM2_Supporting+PP3_Moderate).

Variant 2 is c.1358A>G (Figure 3B): this missense variant was a pathogenic variant which has been reported in previous literature and recorded in Clinvar Database (PM3_Very strong). It had not been recorded in dbSNP and 1,000 genome Project, with minor allele frequency (MAF = 0.0001134) from gnomAD. (PM2_supporting). Both sift and Polyphen2_HDIV software predicted the variant to be deleterious (PP3_moderate). ACMG judged this variant was pathogenic (PM3_Very strong + PM2_Supporting + +PP3_Moderate).

**FIGURE 4:**
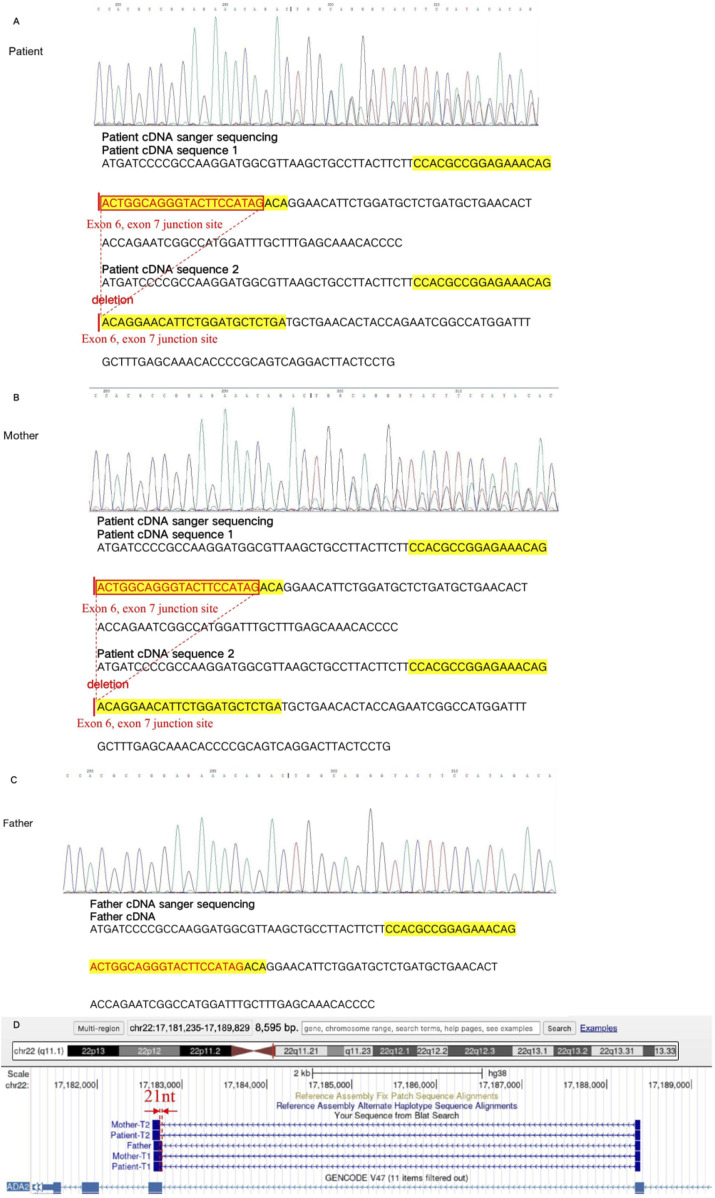
The mRNA reverse transcription-Sanger sequencing in the variant c.1082-7T>A. The cDNA sequence 1 of the patient showed the variant c.1082-7T>A, which led to 21 nucleotides deletion in the exon6, exon 7 junction site, compared with the cDNA sequence 2 **(A)**. The cDNA sequence 1 of mother showed the variant c.1082-7T>A, which leaded to 21 nucleotides deletion in the exon6, exon 7 junction site, compared with the cDNA sequence 2 **(B)**. The cDNA sequence of father showed the normal result **(C)**. The variant c.1082-7T>A was finally confirmed with the affect on mRNA splicing. It lead to 21 nucleotides deletion in the exon6, exon 7 junction site, resulting, finally resulting protein truncation Leu362Glnfs*45 **(D)**.

To further confirm the effect of the variant c.1082-7T>A on protein change, we performed mRNA reverse transcription-Sanger sequencing. Finally, we confirmed that this variant can affect protein splicing, resulting in a frameshift with premature stop codon, r.1083_1103del p. (Leu362Glnfs*45). The RNA study allowed the application of the PVS1_strong ACMG pathogenicity criterion, and the reclassification of the variant as likely pathogenic. The deletion was locadted in the exon 6, exon 7 junction site (Figure 4).

**FIGURE 5:**
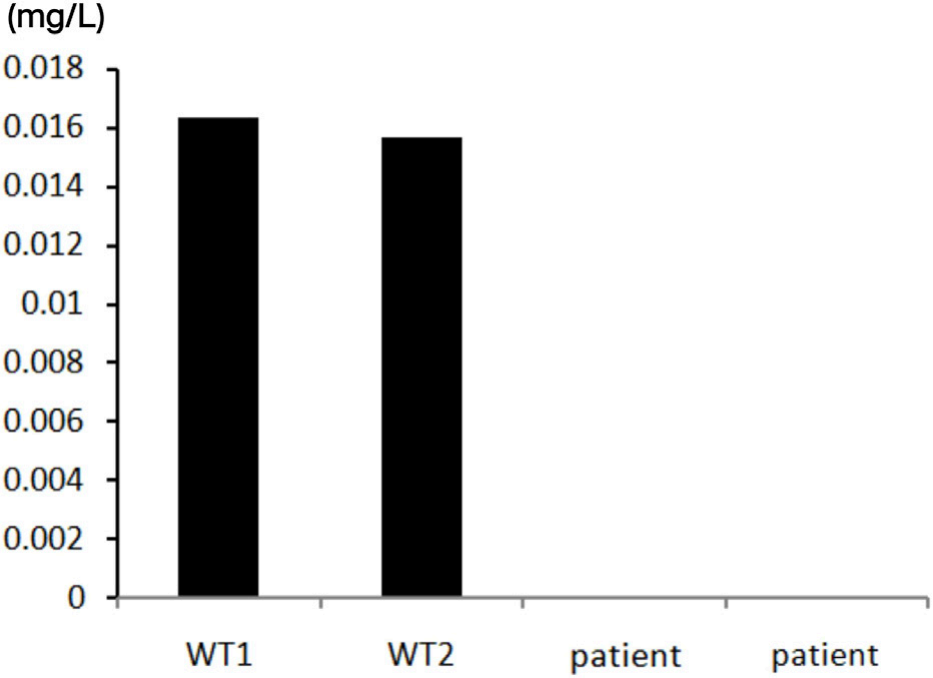
The test of ADA2 enzymatic activity of the patient. Compared with the wild type individuals, the patient’s ADA2 enzyme activity showed a significnat decrease. The ADA2 enzyme activity was 0 (Normal value range >0.003 mg/L).

At the same time, we performed enzymatic examination. In the presence of adenosine substrate, adenosine deaminase catalyzes it to creatinine and releases ammonia. In the presence of NADH, ammonia reacts with α-ketoglutarate catalyzed by glutamate dehydrogenase (L-GDH). Specific experimental method: Inhibited the enzymatic activity of ADA1 by adding EHNA(erythro-9-(-laydfoxy-3*nonyl)adeninel), incubated the reaction system at 37°C for 2 h, measured the absorption peak at a wavelength of 560 nm every 2 min, and quantified the consumption rate of NADH in the system by calculating its slope. This slope was the ADA2 enzymatic activity. The patient’s ADA2 enzyme activity was 0. The specific results are shown in Figure 5.

Our patient was relatively young at disease onset with delayed mental and motor development and showed persistent fever, systemic inflammation, and small vasculitis in the central nervous system. Multiple systems were also involved, such as the blood system and the immune system. Her unique clinical manifestations made us consider inherited diseases. Trio WES finally confirmed the compound heterozygous variants of *ADA2*, and the result of ADA2 enzyme activity assay helped in the final diagnosis of DADA2.

Based on the previous research, we considered that tumor necrosis factor-α antagonist therapy is a good choice. A weekly dose of 12.5 mg etanercept was given subcutaneously. The girl’s body temperature has continued to be normal after treatment, with blood routine examination and CRP levels showing normal results. Brain MRI showed that the lesions had improved (Figure 2). Muscle strength in the left limb recovered to grade IV. The patient was followed-up for 3 years, during which time no fever or hemiplegia recurred.

## 3 Discussion

Deficiency of ADA2 is a rare, autoinflammatory disease, which mainly involves small and medium arteries. There is no formal diagnostic standard at present. DADA2 should be suspected in patients with autoinflammatory diseases characterized by vasculitis, immune dysfunction, and abnormal blood system. Its clinical manifestations are diverse, and it may involve multiple systems (Batu et al., 2015). ADA2 is a growth factor required for differentiation of endothelial cells and white blood cells, which can catalyze the conversion of adenosine into inosine. The lack of ADA2 impairs the integrity of endothelial cells, while at the same time, the process of monocytes transforming into immunomodulatory M2 macrophages (anti-inflammatory) is impaired, resulting in the increase of M1 cells (pro-inflammatory) and promoting the inflammatory response (Moore and Tabas, 2011; Woollard and Geissmann, 2010; Caorsi et al., 2016). Typical symptoms and signs of DADA2 include vasculitis (rash, stroke may occur if the central nervous system is involved); systemic inflammation (manifested by stunting in children, fever, enlarged lymph nodes, and hepatosplenomegaly); bone marrow failure (anemia, neutropenia, Evans syndrome, idiopathic thrombocytopenic purpura); and immune deficiency (low immunoglobulin level) (Zhou et al., 2014; Navon et al., 2014; Ben-Ami et al., 2016; Sundin et al., 2019).

The clinical manifestations of DADA2 are characterized by systemic inflammatory response and multiple vascular lesions, and the systemic inflammatory response and rash are prominent and typical changes, which were easily misdiagnosed as polyarteritis nodular in the past, and have also been called “hereditary polyarteritis nodosa.” The onset of this disease is usually in childhood, with 24% cases reporting onset before the age of 1 year, and 77% reporting onset before the age of 10 years (Wang et al., 2021a). At present, More than 316 cases with diverse clinical phenotypes have been reported, with the most common affected systems being the skin and central nervous system. Among these, 90% of cases have reported skin involvement. Characteristic cutaneous vascular lesions are manifested as livedo reticularis and erythematous nodules (Lee, 2018; Ombrello et al., 2019). Neurological involvement is mostly manifested as cerebral ischemic stroke and common lacunar infarction, with the brainstem and deep gray matter nuclei being affected, especially the punctate brainstem localized DWI hyperintensity, which is a typical imaging change. The gastrointestinal tract, liver, kidneys, and coronary system are also affected to varying degrees (Lee, 2018).

The gene encoding ADA2 is a highly polymorphic gene, and DADA2-related variants are located in the entire coding region of *ADA2*, including more than 300 missense and deletion variants (Meyts and Aksentijevich, 2018). Most patients’ variants were compound heterozygous missense variants, with the most common disease causing variants in *ADA2* gene being p. Gly47Arg, p. Gly 47Ala, p. Arg169Gln and p. Tyr453Cys (Zhou et al., 2014; Navon et al., 2014).

This patient had early onset at age 2 years and 3 months and the main clinical manifestations were fever, hemiplegia, and transient rash. Upon admission, the hemoglobin level was low, CRP level ESR were significantly increased, and immunoglobulin IgA and IgM were lower than normal. After initially excluding infectious diseases, the manifestations of multisystem involvement (including the skin, blood, nervous system, and immune system) prompted us to primarily consider autoinflammatory or autoimmune disorders. Although the skin changes observed in this case are atypical, the prominent systemic inflammatory response and the characteristic ischemic lesions of multiple small blood vessels in the brain provided important diagnostic clues. Considering the young age of onset and delayed development, we were inclined to make a diagnosis of inherited vasculitis (Wang et al., 2021b). Subsequently, she was confirmed to have DADA2 through genetic and enzymatic testing. Based on the pathogenesis and existing literature, we concluded that tumor necrosis factor-α (TNF-α) antagonist therapy was an appropriate treatment option (Ombrello et al., 2019; Hashem et al., 2017; Caorsi et al., 2017). Following treatment with etanercept, the patient’s condition showed significant improvement.

In conclusion, this is the first report of the c.1082-7T>A gene variant in a patient with DADA2, thereby expanding the genetic variant profile of the *ADA2* gene. Notably, the RNA study performed provides strong evidence supporting the pathogenicity of this variant, further strengthening the findings of this report. The clinical phenotype of DADA2 is complex, and differential diagnosis can be challenging. The patient’s prominent symptoms included persistent fever and hemiplegia, without the typical rashes, which may lead to the disease being overlooked. The significant inflammatory factors and typical MRI changes combined with multiple systems involvement, led to suspicion of this rare disease. The trio-WES and enzyme activity assay can help clinicians make the right diagnosis. For children with a clear systemic inflammatory response and symptoms of vasculitis involving the central nervous system, especially those with an early onset, it is important to consider both common immune-related vasculitides and the possibility of vasculitis due to genetic variants. Genetic testing may be necessary for an accurate diagnosis. A correct diagnosis is crucial for timely treatment, which can have significant benefits for these patients.

## Data Availability

All relevant data is contained within the article. The original contributions presented in the study are included in the article, further inquiries can be directed to the corresponding authors.
